# Spatial patterns and associated factors of HIV Seropositivity among adults in Ethiopia from EDHS 2016: a spatial and multilevel analysis

**DOI:** 10.1186/s12879-020-05456-y

**Published:** 2020-10-14

**Authors:** Bayuh Asmamaw Hailu, Fentaw Tadese, Getahun Gebre Bogale, Asressie Molla, Birhan Asmame Miheretu, Joseph Beyene

**Affiliations:** 1 Department of Epidemiology and Biostatistics, School of Public Health, College of Medicine and Health Sciences Wollo University, Dessie, Ethiopia; 2 Department of Health Informatics, School of Public Health, College of Medicine and Health Sciences, Wollo University, Dessie, Ethiopia; 3 Department of Geography and Environmental Studies, Wollo University, Dessie, Ethiopia; 4 Department of Health Research Methods, Evidence, and Impact, McMaster University, Hamilton, Canada

**Keywords:** HIV seropositive, AIDS, Adult, Multilevel, Spatial

## Abstract

**Background:**

HIV is a major public health issue, especially in developing countries. It is important to track and design successful intervention programs to explore the spatial pattern, distribution, and associated factors of HIV Seropositivity. This study therefore showed the spatial variation of HIV Seropositivity and related factors in Ethiopia.

**Methods:**

A total sample of 25,774 individual data collected from the 2016 EDHS data were primarily HIV biomarkers, IR, MR, and GPS. Spatial heterogeneity analysis was used with methods such as Morans I, Interpolation, and Kulldorff ‘s scan statistic. Spatial analysis was conducted using open source tools (QGIS, GeoDa, SaTScan). Multilevel logistic regression analysis was performed using Stata14 to identify HIV-associated factors. Finally, the AOR with a 95% confidence interval was used to report the mixed-effect logistic regression result in the full model.

**Result:**

The prevalence of HIV / AIDS at national level was 0.93%. The highest prevalence regions were Gambela, Addis Abeba, Harari and Diredawa, accounting for 4.79, 3.36, 2.65 and 2.6%, respectively. Higher HIV seropositive spatial clusters have been established in the Gambela and Addis Ababa regions. Multilevel analysis at the individual level being married [AOR = 2.19 95% CI: (1.11–4.31)] and previously married [AOR = 6.45, 95% CI: (3.06–13.59)], female [AOR = 1.8, 95% CI: (1.19–2.72)], first-sex at age ≤15 [AOR = 4.39, 95% CI: (1.70–11.34)], 18—19 [AOR = 2.67 95% CI: (1.05–6.8)], middle age group (25-34) [AOR = 6.53, 95% CI: (3.67–11.75)], older age group (>34) [AOR = 2.67 95% CI: (1.05–6.8)], primary school [AOR = 3.03, 95% CI: (1.92–4.79)], secondary school [AOR = 3.37, 95% CI: (1.92–5.92) were significantly associated with serropositivity. Regarding household level, place of residence [urban: AOR = 6.13 CI: (3.12, 12.06)], female-headed households (AOR = 2.24 95% CI: (1.57–3.73), media exposure [low exposure (AOR = 0.53 95% CI: (0.33–0.86), no exposure AOR = 0.39 95% CI: (0.23–0.65)] and increased household size [AOR = 0.72 95% CI: (0.65–0.8)] were associated with HIV Seropositivity.

**Conclusion:**

High cluster HIV cases were found in Gambela, Addis Abeba, Harari, and Diredawa. Having a history of married, start sex at a younger age, female-headed household, urban residence, and lower household size is more affected by HIV/AIDS. So any concerned body work around this risk group and area can be effective in the reduction of transmission.

## Background

Acquired Immune Deficiency Syndrome (AIDS) is a global epidemic caused by a virus called the Human Immune Deficiency Virus (HIV). It affects the body’s human immune system. The outbreak was first identified in 1980. The key mode of transmission is unprotected sexual activity, although different in different regions of the world. For example, homosexual sex and intravenous drug injection are commonly assumed to be the means of transmission in developed countries. Heterosexual communication, on the other hand, is the dominant mode of transmission in developing countries [1].

Every country is affected by the HIV epidemic. An estimated 0.8% of adults worldwide have been living with HIV, although the burden of the epidemic varies considerably between countries and regions being higher in developing countries, particularly Sub-Saharan Africa (SSA). According to recent UNAIDS (United Nations Program on HIV/AIDS) estimates, individual HIV/AIDS epidemic prevalence is less than 2% in many West and Central African countries [2].

Globally, 37.9 million people were infected with HIV; 1.7 million new HIV infections (incidence), and 770,000 HIV and HIV-related deaths occurred in 2018. The majority of cases have occurred in Eastern and Southern Africa [3]. Approximately 5000 HIV infections occur every day in the world, of which 61% are in SSA [3].

Ethiopia is barely affected by this deadly pandemic, and a substantial number of sick people have been living with HIV. HIV prevalence among Ethiopian was reported (1.3%) in 2010, in 2015 (1.1%) and then on 2018 (1%) [3]. In Ethiopia, the annual HIV prevalence rate decreased from 3.3% in 2000 to 1% in 2018, and AIDS-related deaths from 83,000 in 2000 to 11,000 in 2018 [3, 4].

Efforts to reduce HIV infections are off-track; the incidence rate of HIV is decreasing every year and the 2020 target (fewer than 500,000 new infections) was set. While reductions in HIV/AIDS-related deaths are good, the reduction goals may be missed [3].

HIV / AIDS is widespread among adults (working age) [5]. Long-term illness due to HIV / AIDS needs higher medical costs for HIV-affected households. HIV/AIDS decreases savings and productive assets and raises the liability of HIV-affected households [6]. The household and community were higher medical expenditures, which is the consequence of lack of healthy food, reduce investment in farming and industry, and affect children’s education [7].

Measures of disease occurrence are usually seen only by large geographical administrative units. Big, sparsely populated geographical areas (national and regional) can mask geographical heterogeneity and could potentially cause a misinterpretation of the true underlying geographical patterns [8].

The pattern and distribution of HIV in the country differ widely due to the current socio-cultural diversity of Ethiopia. Recent studies suggest that there is a significant difference in HIV prevalence across regions of the world and the place of residence [9, 10].

However, the majority of previous studies in Ethiopia concentrated on prevalence and disease-related factors at the individual level, using a small sample size and some particular areas [11–13].

Geographical space plays a role in the identification of populations at higher risk. This main aspect of the outbreak has also been poorly explored in the sense of HIV. This study is carried out using 2016 EDHS to examine the spatial trends and determinants of HIV Seropositivity in Ethiopia, using geographic analysis techniques to classify risk at the regional or country level, in addition to factors associated with different and closest nesting levels (household and individual rather than regional).

## Methods

### Study area

The study was conducted in Ethiopia, situated in the North-Eastern part of Africa. It is bounded by North and South Sudan in the West, by Eritrea and by Djibouti in the North East, Somalia only in East and South East, and by Kenya in the South. Ethiopia is located between 3^0^ N and 15^0^ N Latitude and 33^0^ E and 48^0^ E Longitude.

The country covers an area of approximately 1,127,000 km^2^. The Ethiopian landmass consists of a large, high plateau that crosses the Rift Valley into the northwest and southeastern highlands, each with its associated lowlands. The difference in relief is striking as land elevation varies from 130 metres below sea level (Dallol depression located in Afar region) to Moutain Ras-Dashn peak at 4620 meters above sea level in the Semen Mountains [14]. There are nine regional states and two city administrations.

### Study design

A population-based cross-sectional study was used to analyze HIV seropositivity, to investigate the spatial distribution of HIV, and to identify HIV-related factors in Ethiopia.

### Data source

The data for this analysis was taken from the 2016 EDHS, which is the fourth comprehensive and nationally representative survey conducted in Ethiopia, as part of the Global Demographic and Health Surveys (DHS). EDHS 2016 data has been downloaded from the DHS website after permission has been given.

### Dependent variables

#### HIV Seropositivity (positive, negative)

The interviewer collected capillary blood from a finger prick in women between 15 and 49 years of age and men between 15 and 59 years of age who consented to HIV testing. The blood sample collection and analysis protocol was based on the anonymous unlinked protocol developed for the DHS program. If the respondent consented to HIV testing, five-spot of blood from the finger pin were obtained on a filter card. A specific barcode label was affixed to the filter slip, a duplicate label was attached to the biomarker questionnaire, and a third copy of the same barcode was affixed to the Dried Blood Spot Transmittal Sheet to monitor blood samples from the field to the laboratory. Blood samples were dried overnight and packed for storage the following morning. Samples were periodically collected from the field and transported to the laboratory of the Ethiopian Public Health Institute (EPHI) in Addis Abeba. Upon arrival at EPHI, each blood sample was logged into the CSPro HIV Test Tracking System database with a laboratory number and stored at-20 °C until checked [15].

### Sample size and sampling procedures

A total of 25,774 individuals out of which 13,295 women and 12,479 men were included in the analysis from 13,043 households. All sampling procedures, data collection, and data quality control were done by the DHS team [16].

### Data processing and analysis

Data from 25,774 individuals were collected from the 2016 EDHS and analyzed using Stat 14. Descriptive statistics have been calculated using frequency and proportion.

### Spatial statistics analysis

Statistically significant clusters are characterized as geographical areas where the prevalence of the disease is disproportionately higher / lower compared to neighboring areas. The global clustering test detect the presence of at least one cluster, but not the precise location of the cluster(s).

### Mapping cluster

Mapping clusters were done using the Local Indicators of Spatial Association (LISA) analysis and GeoDa was used to conduct LISA analysis. LISA measures spatial autocorrelation, a measure of the degree to which features clustered or dispersed, and can be used as a method for cluster analysis. In cluster analysis objects in the same group (cluster) are more similar to each other than others [16, 17].

Local Moran’s I used to map disease prevalence clusters, and classify major clusters. There are four groups, the clusters are high (high disease prevalence rates whose neighbors also have high prevalence rates) and low (lower infection prevalence whose neighbors also have low prevalence rates) areas. This showed positive spatial autocorrelation and clarified clusters, while remaining clusters were outliers.

### Interpolation

Interpolation analysis was performed using QGIS and is based on the assumption that spatially distributed objects are spatially correlated. I.e. objects that are close together appear to have similar characteristics or, depending on the measured area, estimate undetermined area by using the Inverse Distance Weight (IDW) [18–20].

### Kulldoruff’s scan statistic

Kulldoruff ‘s scan statistic is a tool that uses spatial scan statistics for the identification and assessment of statistically important spatial cluster risk factors for a particular disease. The final confirmatory spatial analysis was performed using SaTScan with QGIS analysis tools. The SaTScan may distinguish particular locations were higher or lower rate of spatial aggregate. Its output presents the hotspot areas in circular windows, indicating areas of windows are higher than expected distributions compared to the areas outside of the cluster windows [21–24].

### Multilevel logistic regression

Multilevel analysis was considered appropriate to take into account the hierarchical nature of the DHS data and to be able to estimate both individual-level and household effects on the outcome variable [25–27].

### Model building

Multilevel analysis was performed using Stata 14. The analysis was carried out in four stages. The first model (M0) is an empty model fitted without independent variables to test random variability [28]. The second model (M1) was tailored to individual factors; the third model (M2) was used for household-level factors; and the fourth model (M3) was used for both individual-level and household-level factors. Akaike Information Criterion (AIC) model fitness for the report was selected.

## Results

A total of 25,774 adult participants have been included in the study. Women have been beyond half of 13,295 (51,58%). As for age 9536 (37%) younger (15—24). The majority of rural residents 20,368 (79%), and 21,094 (82%) of male households. Regarding educational status, 10,130 (39%) of respondents did not pursue formal schooling.

The overall risk of HIV seropositivity in Ethiopia was found to be 0.93% (*n* = 239) with 95% confidence interval from 0.8 to 1.0%. The proportion of HIV seropositivity among males was 0.29% (*n* = 74) while 0.64% (*n* = 165) among females. Geographical distribution of HIV seropositivity.

**Fig. 1:**
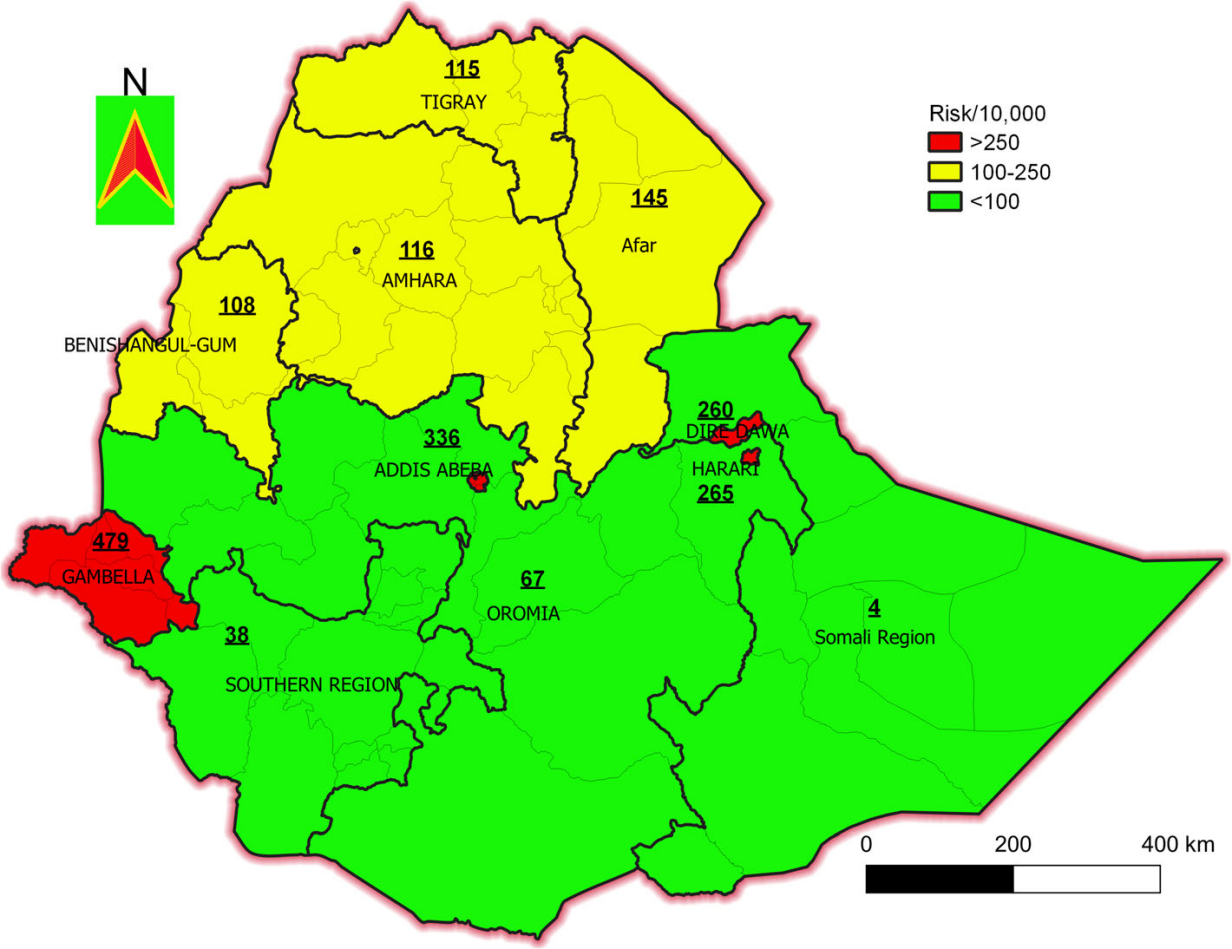
Regional risk of HIV Seropositive per 10,000 adults in Ethiopia. The green color indicates the risk of HIV below 100 per 1000; The yellow color indicates the risk of HIV between 100 and 250 per 1000; The red color indicates the risk of HIV above 250 per 1000 adults in Ethiopia. Additionally, the risk of HIV per 1000 people in each region is put on their site. This analysis is carried out QGIS 3.12.2 which available at https://www.filecroco.com/download-quantum-gis/download/

Regional HIV seropositive from the highest to the lowest was Gambella (*n* = 479), Adis Ababa (*n* = 336), Harari (*n* = 265), Diredawa (*n* = 260), Afar (*n* = 145), Amhara (*n* = 116), Tigray (*n* = 115), Benishangul Gumz (*n* = 108), Oromia (*n* = 67), Southern Nation Nationality People (SNNP) (*n* = 38) and Somalia regions (*n* = 4) (Fig. 1).

### Mapping cluster

**Fig. 2:**
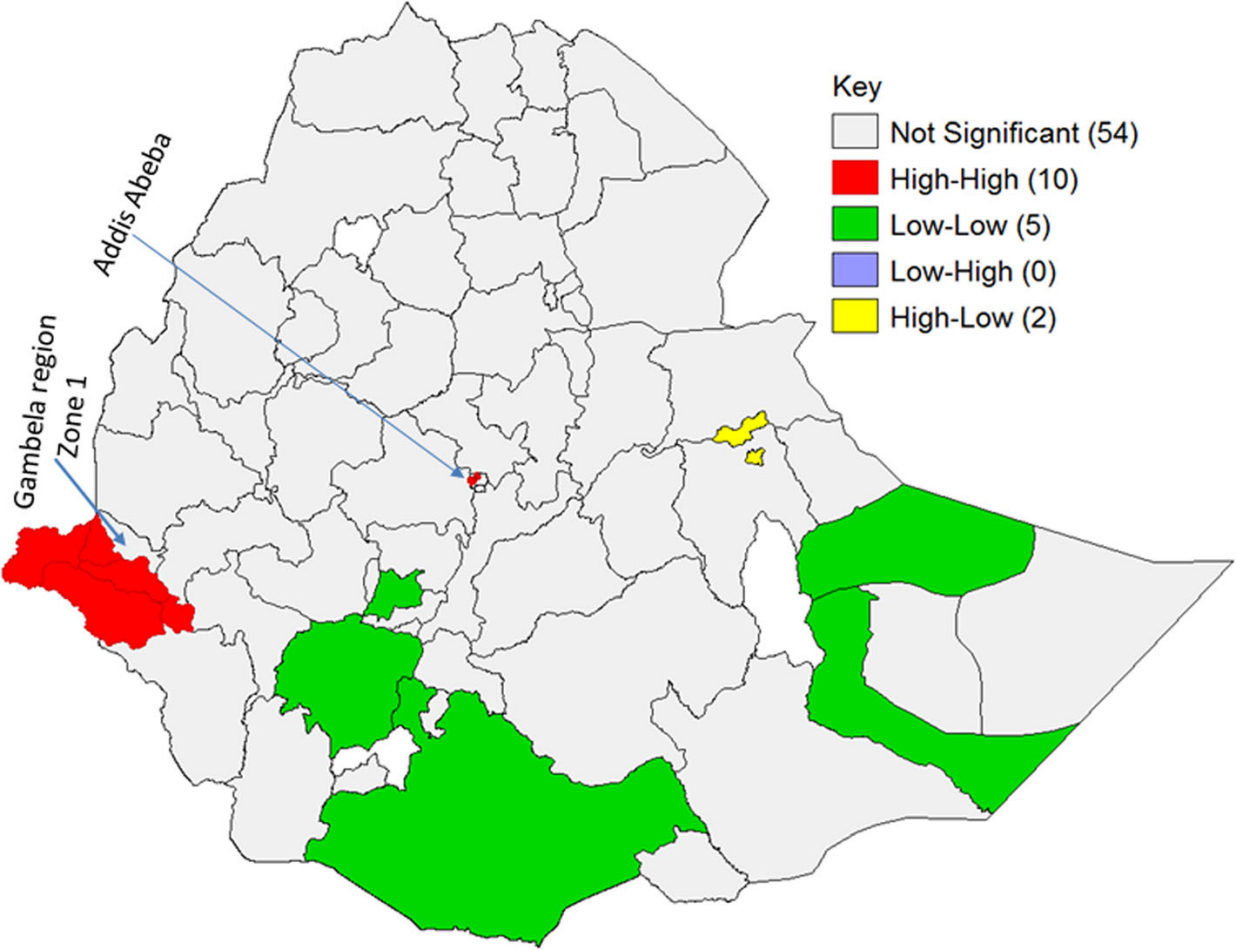
LISA cluster map of HIV Seropositivity in Ethiopia. Each polygon on the map represents a single zone area with a burden of HIV/AIDS. High-High (red color) means high rates of HIV/AIDS surrounded by similar characteristics; low-low (green color) means low rates of HIV/AIDS surrounded similar characteristics. High-low (yellow color) means high rates of HIV/AIDS surrounded by low rates of HIV/AIDS in adults; undefined (white color) indicates lakes and zones (not taken sample in this area). The red color indicates hotspot areas of HIV/AIDS; the green color indicates cold-spot areas of HIV/AIDS; and the yellow and dark-blue colors show outliers. The hotspots are public health importance. To conduct this analysis use GeoDa version 1.14, which is available at: https://geodacenter.github.io/download_windows.html

The LISA cluster map showed *p* < 0.001 with Moran’s I being 0.432. The Moran I indicated cluster patterns (Fig. 2).

The red color shows a high proportion of HIV seropositivity, surrounded by high rates of similar cases. These specific areas were Adis Ababa and areas of Gambela except Zone 1 at the border of the Oromia region (Fig. 2).

#### Spatial interpolation

**Fig. 3:**
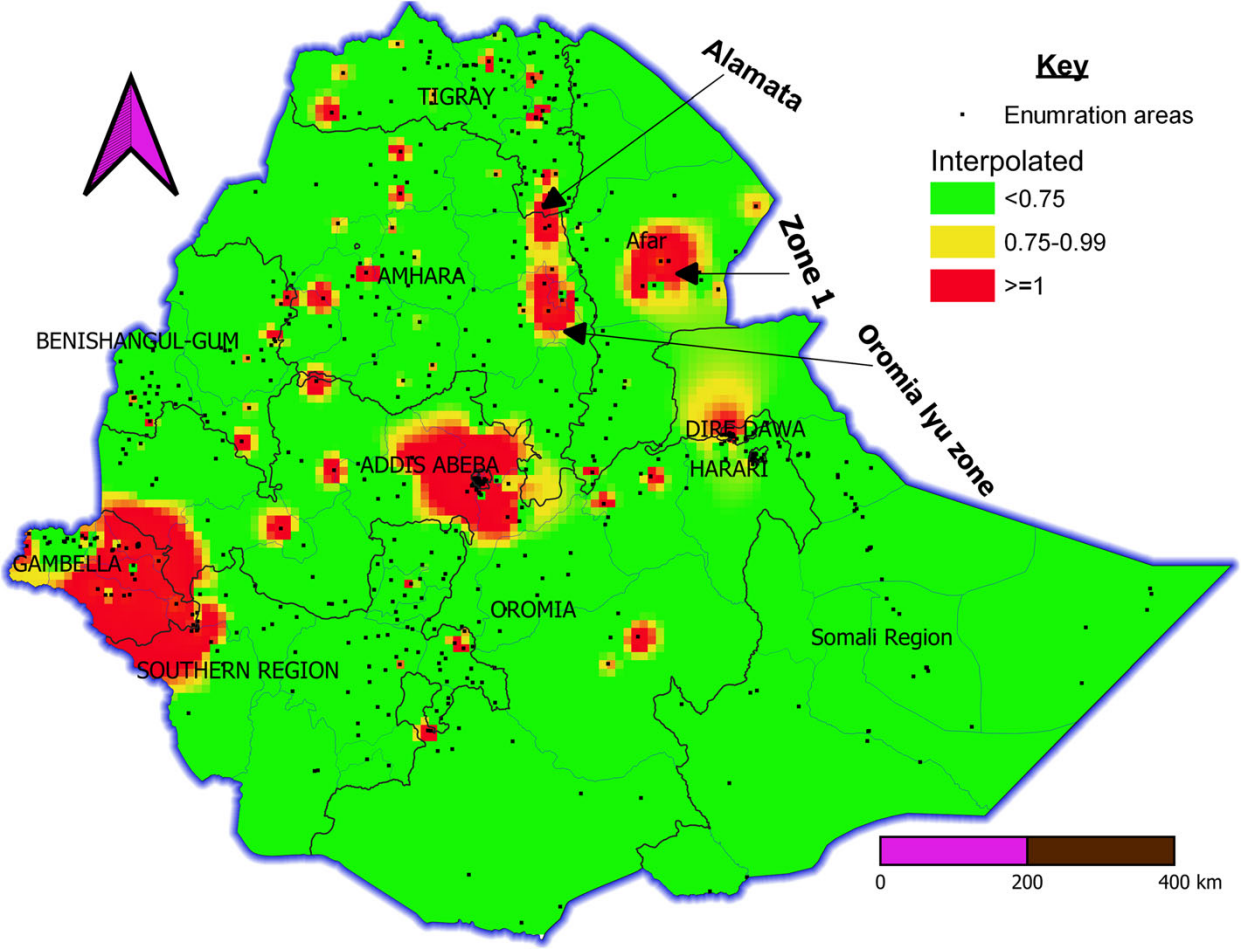
IDW interpolation HIV Seropositive prediction map in Ethiopia. Interpolated Continuous images produced by interpolating Inverse Distance Weight (IDW) HIV/AIDS among adults in Ethiopia. The red ramp color indicates the predicted HIV/AIDS high-risk areas and the green color indicates less risk areas of HIV seropositivity. The black point indicates enumeration area This analysis is carried out QGIS 3.12.2 which available at https://www.filecroco.com/download-quantum-gis/download/

In spatial interpolation, the forecast showed high-risk areas (red color), Gambela region and its surrounding areas, Addis Ababa and its surrounding areas, Diredawa, Afar Region Zone 1 at the direction of Djibouti, and Amhara Region from Oromia Liyu zone to Alamata via cross country roads (main road) and sporadic in all regions except Somalia region (Fig. 3).

### Kulldoruff’s scan statistic

**Fig. 4:**
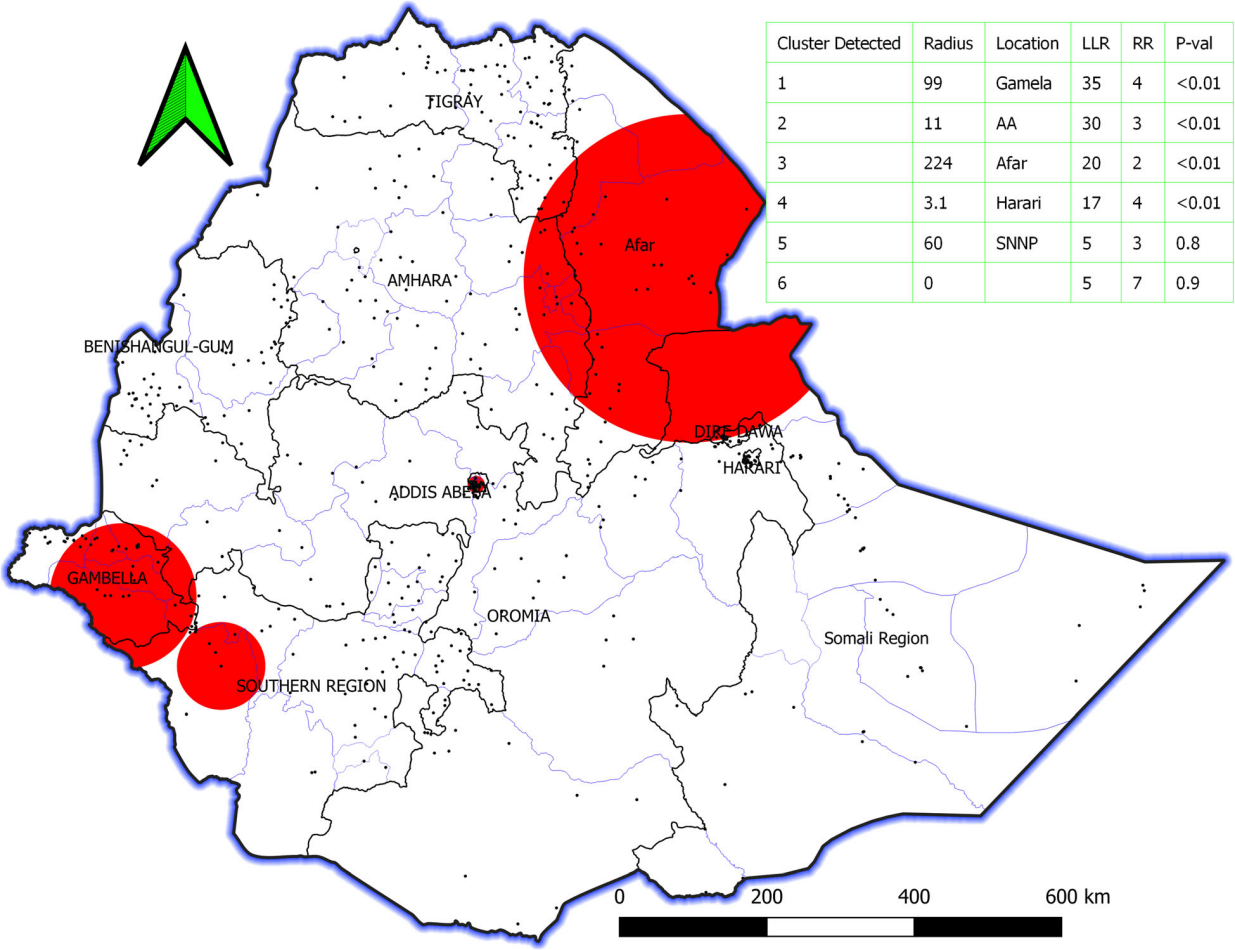
HIV Seropositivity hotspot clusters identified using SaTScan spatial analysis tool, in Ethiopia. Te red color circle indicates hotspot windows. And the dot indicates the enumeration cluster. The table legend identifies statistically significant (*p*-value), location (site), relative risk, log-likelihood ratio. To conduct this analysis use SaTScan v9.6, which is available at https://www.satscan.org/download.html and QGIS 3.12.2

The most likely primary spatial clusters for HIV were identified by SaTScan. A total of 6 clusters were observed by order from the SaTScan spatial analysis and only 4 clusters were statistically significant circular windows. These were all areas of Gambela, Addis Ababa, most of Afar, Harari, Somalia at the border of Afar, and Amhara at the border of Afar (Fig. 4).

### Multilevel logistic regression

#### Model comparison for report

The following table summarizes the fitness for multilevel logistic regression models (Table 1). The fourth model (M3) is the final best model, which incorporates both individuals with household characteristics simultaneously, which is the only model included in the report. Since the lowest of the AIC and the highest of the log-likelihoods is the best. If IHHC indicates that > 5% differs between households (Table 1).

**Table 1:** Model selection for report

Model	IHHC	AIC	Log-likelihood
M_0_	**42.74%**	−36,002	18,003.8
M_1_	42.02%	−36,779	18,408.7
M_2_	41.87%	−36,381	18,200.7
M_3_	41.66%	**−36,974**	**18,512.8**

Adjusted for the individual and household level variables (model 3) sex of respondents, age, level of education, marital status, first-sex age, female-headed household, media exposure, residence, and household size were significantly associated with risk of HV seropositivity.

The female-headed household had 2.42 times higher prevalence of infection than the male-headed household (AOR = 2.42, 95% CI 1.57–3.73). Besides, females were 80% more likely to be infected with HIV than males (AOR = 1.8, 95% CI: 1.19—2.72). Individuals in the middle age group(25 to 34) or older (above 34) age were 4.75 and 4.33 times higher HIV seropositivity compared to young (below 25) age groups with (AOR = 6.57, 95% CI: 3.67–11.75) and (AOR = 14.5, 95% CI: 7.73–27.35) respectively.

HIV seropositivity for married (living togather) and previously married (divorced or widowed or separated) were 2.19(AOR = 2.19, 95% CI: 1.11–4.31) and 6.45 (AOR = 6.45, 95% CI:3.06–13.59) respectively, times HIV seropositive than unmarried individuals. Media exposure was low exposure 47% (AOR = 0.53, 95% CI: 0.33—0.86) and no exposure 61% (AOR = 0.39, 95% CI: 0.23—0.65) lower than that of high media exposure respondents.

Regarding the educational level of individuals who had primary and secondary levels of education were 3.03 and 3.37 times most likely to be infected by HIV than those who had no formal education (AOR = 3.03, 95% CI: 1.92–4.79) and (AOR = 3.33, 95% CI:1.92–5.92), respectively. The study participants from urban households were 6.83 times more likely to be infected with HIV than rural households (AOR = 6.83, 95% CI: 3.11—14.99).

Looking at the age at first sex, individuals who began sex less than 16 years of age, and 18–19 years of age were 4.39 and 2.67 times more likely to be infected with HIV (AOR = 4.39. 95% CI: 1.70–11.34) and (AOR = 2.67. 95% CI: 1.05–6.80) respectively than those who never began sex. When the household size rose by one family member, HIV / AIDS seropositivity decreased by 28% (Table 2).

**Table 2:** The distribution and multilevel logistic regression analysis of factor associated with HIV Seropositivity in Ethiopia, 2016

Characteristics	Frequency (%)	N**o** Positive (%)	COR(95%CI)	AOR(95%CI)
Total	25,774 (100)	239 (0.93)		M3
***Individual-level***				
Marital status				
Single	8119 (31.50)	24 (0.30)	1	1
Living together	16,132 (62.59)	135 (0.84)	3.93 (2.3,6.73)	2.19 (1.11,4.31)**
Separated	1523 (5.91)	80 (5.25)	48.55 (25.12,93.83)	6.45 (3.06,13.59)**
Sex				
Male	12,479 (48.42)	74 (0.59)	1	1
Female	13,295 (51.58)	165 (1.24)	2.46 (1.76,3.44)	1.8 (1.19,2.72)**
Age at first sex				
No had sex	6776 (26.29)	12 (0.18)	1	1
> 20	6932 (26.90)	54 (0.78)	8.95 (4.13,19.43)	1.32 (0.52,3.38)
18—19	3614 (14.02)	51 (1.41)	18.51 (8.23,41.62)	2.67 (1.05,6.8)**
16—17	3652 (14.17)	44 (1.20)	9.91 (4.39,22.38)	2.16 (0.83,5.63)
< 15	4800 (18.62)	78 (1.63)	11.43 (6.38,18.38)	4.39 (1.70,11.34)**
Working status				
Have work	10,293 (39.94)	78 (0.76)	1	1
Haven’t work	15,481 (60.06)	161 (1.04)	1.41 (1.01,1.97)	1.11 (0.76,1.61)
Age				
Younger	9536 (37.00)	19 (0.20)	1	1
Middle	8055 (31.25)	81 (1.01)	8.1 (4.25,15.4)	6.53 (3.67,11.75)**
Older	8183 (31.75)	138 (1.69)	16.8 (9.01,31.35)	14.5 (7.73,27.35)**
Education level				
Uneducated	10,130 (39.30)	56 (0.55)	1	1
Primary	10,547 (40.92)	114 (1.08)	1.9 (1.27,2.85)	3.03 (1.92,4.79)**
Secondary	3310 (12.84)	50 (1.51)	3.37 (2.02,5.6)	3.37 (1.92,5.92)**
Higher	1787 (6.93)	19 (1.06)	1.83 (0.92,3.66)	1 (0.5,2)
Knowledge				
Have knowledge	9365 (39.98)	106 (1.13)	1	1
Haven’t knowledge	14,061 (60.02)	121 (0.86)	0.76 (0.54, 1.05)	0.91 (0.66,1.27)
Age at first mirage				
> 20	7737 (43.82)	88 (1.14)	1	
18—19	2763 (15.65)	39 (1.41)	1.26 (0.76,2.22)	
16—17	2850 (16.14)	30 (1.05)	0.95 (0.53,1.68)	
< 15	4305 (24.38)	58 (1.35)	1.25 (0.78,2.01)	
Circumcise				
No	1002 (8.05)	5 (0.50)	1	
Yes	11,445 (91.95)	69 (0.60)	0.51 (0.15,1.83)	
***Household-level***				
Media exposure				
High exposure	8199 (31.81)	145 (1.77)	1	1
Low exposure	5675 (22.02)	40 (0.70)	0.37 (0.23,0.58)	0.53 (0.33,0.86)**
No exposure	11,900 (46.17)	54 (0.45)	0.2 (0.13,0.3)	0.39 (0.23,0.65)**
Residence				
Rural	20,368 (79.03)	86 (0.42)	1	1
Urban	5406 (20.97)	153 (2.83)	17.68 (10.8,28.95)	6.83 (3.11,15)**
Sex of household head				
Male	21,094 (81.84)	126 (0.60)	1	1
Female	4680 (18.16)	113 (2.41)	6.2 (4.1,9.39)	2.24 (1.57,3.73)**
Size of household	25,774 (100)	239 (0.93)	0.66 (0.6,0.73)	0.72 (0.65,0.8)**
Wealth of household				
Poorest	4217 (16.36)	21 (0.50)	1	1
Poorer	4691 (18.20)	11 (0.23)	0.55 (0.21,1.45)	0.45 (0.2,1.01)
Middle	4945 (19.19)	17 (0.34)	0.64 (0.25,1.61)	0.55 (0.25,1.22)
Richer	5292 (20.53)	34 (0.64)	1.38 (0.61,3.08)	0.8 (0.37,1.61)
Richest	6629 (25.72)	156 (2.35)	11.7 (5.75,23.88)	0.55 (0.23,1.32)

The odds ratios were adjusted for all other variables constant in the respective models. ** Significant at *P*-value < 0.05

## Discussion

The overall HIV/AIDS prevalence in Ethiopia was 0.93%; lower than in other countries, such as Uganda, 6.5%, Kenya 5.4%, Rwanda 3.1%. On the other hand, this finding is found to be higher than studies from Somalia 0.4%, Sudan 0.2%, and Chile 0.5% in 2016. The disparity may be a variation in the socio-demographic status of individuals across countries.

The highest top four HIV seropositive region Gambella, Addis Abeba, Harari, and Diredawa. The next highest risk is Northern, Northwest, and Northeast areas of the country, and most of the findings are consistent with the 2011 EDHS finding [15].

Almost all the spatial results suggest that the highest risk zones in the Gambela region have been identified. This result is consistent with other results in the DHS report [29]. The key explanation may relate to current cultures that do not recognize male circumcision at all. Literature suggested that uncircumcised males were at higher risk of HIV compared to their counterparts [30]. Another reason is related to the existence of polygamy practices in the culture. And the lack of infrastructure such as education, health facility that could contribute to low HIV understanding.

The next highest risk of HIV in Addis Abeba. This finding is similar to that of the EDHS report [29]. Which is the largest city in the country and the main city of the African Union, and has various industries, one of the reasons in this area is the higher number of commercial sex-worker.

The next high-risk areas were the Diredawa City Administration and the Harari Region. This could be attribute khat chewing practice is higher and culturally accepted at the community level. Besides, a study showed that Khat chewers were at higher risk for HIV [31]. This may a khat in nature diverts a personal attitude, which means people with chewing khat may not care about stuff due to addiction. On the other hand, much of the khat chewing is accompanied by consuming alcohol, which might lead to sexual risk behavior. The remaining high-risk areas are situated in the Afar region on the border of Djibouti and in the Amhara region especially Wollo main road to Tigray. This region has been in touch with high-risk individuals, such as long-distance vehicle drivers and day-to-day workers and higher human traffic [32].

Place of residence was found to be positively associated with HIV seropositivity, as urban residents were more likely to be infected with HIV than their rural counterparts. This result is consistent with other studies [33, 34]. Urban residents may have a high population movement due to trade, labor, and migration. Commercial sex workers and homosexuals individuals live in urban areas considered to be at high risk of HIV infection [3]. The chances of HIV seropositivity were higher as the age of the person increased. This result is consistent with other results [33, 35, 36]. On the other hand, a study done shows the youngest age group predicted new HIV/AIDS infections to spread and unsafe sex due to certain biological and economic reasons [37]. This may be the research measuring the prevalence of HIV seropositivity, which is cumulative from younger to older ages. This is not linked to the age at which the respondents were infected with HIV / AIDS. Individuals who began sex at a younger age were found to be substantially associated with HIV seropositivity.

Individual sex and household-head sex are both predictors of HIV seropositivity, though in both females are at higher risk of HIV seropositive than males. This result is in line with other result. This may be due to the higher biological susceptibility of women to HIV infection [38]. Similarly, female-headed households were more likely to be HIV-positive than male-headed households, and this result is consistent with other studies done in sub-Saharan Africa [39, 40]. In Ethiopia, males are the default household head. However, if the females are the heads of households, it is generally a sign that the marital status of the female is not-married, divorced, or widowed. This may expose women to high-risk sexual activities such as having multiple partners engaged in commercial sex work and witnessing sexual harassment.

Individual education levels was also established as a determinant of HIV seropositivity; people with primary and secondary schools were more likely to have HIV seropositive compared to those without formal education. The results are consistent with other studies [34, 39]. On the other hand, a study reported that HIV prevalence decreases significantly with each increase in education levelthe [31]. One of the reasons for this may be more qualified people to travel around and they can also afford to pay for sex [41, 42].

The marital status of the person was found to be strongly linked to HIV seropositivity. The study found that never marrying people were less likely to have HIV-positive compared to married and previously married people. This result is partly in agreement with another report. A similar finding of previous married is high risk, on the other hand, married higher risk in this finding but low risk in other findings relative to never married [34]. Marital status has a different effect on women and men in many cases due to societal constraints on women’s autonomy in the public sphere. These restrictions may lead to HIV/AIDS, reduce the ability of unmarried women to participate in equal relationships and negotiate healthy sexual practices (condom use), or may live without sex with their partners [43].

The higher prevalence of HIV seropositivity among individuals exposed to high levels of media exposure and is consistent with the SSA study [40].

The odds of HIV being HIV-positive have decreased as the number of household members has grown and is similar to other findings [29]. Having a large family size can cause a tremendous economic burden, and may affect leisure habits that could lead to an individual having a risky sexual behavior [29].

## Conclusion

Minimize the spread of the disease government and any concerned body, use less effort to find more success in the high-risk area and group. High cluster HIV cases have been reported in Gambela, Addis Ababa, Harari, and Diredawa. At the individual level, certain characters are high risk, such as being married before, beginning sex at a younger age, and at the household level, the female head of household, urban residence, and lower family size are more affected by HIV/AIDS. Therefore, anyone operating across this risk group and region may be successful in reducing transmission.

## Data Availability

The data set we used which is the ‘2016 Ethiopian Demographic and Health Survey’ were obtained from the DHS program (www.dhsprogram.com), but the ‘Dataset Terms of Use’ do not permit us to distribute this data as per data access instructions (http://dhsprogram.com/data/Access-Instructions.cfm). To get access to the dataset you must first be a registered user of the website (www.dhsprogram.com) and download the 2016 Ethiopian Demographic and Health Survey datasets.

## Abbreviations

AOR: Adjusted Odds Ratio
CI: Confidence Interval
DHS: Demographic and Health Survey
EDHS: Ethiopia Demographic and Health Survey
IHHC: Intra House Hold Correlation
SNNP: South Nations Nationality People
AIC: Akaike Information Criterion
BIC: Bayesian Information Criterion
LISA: Local Indicators of Spatial Association
AIDS: Acquired Immune Deficiency Syndrome
HIV: Human immune deficiency virus
UNAIDS: United Nations Program on HIV/AIDS
SSA: Sub Sahara Africa
EPHI: Ethiopian Public Health Institute
IDW: Inverse Distance Weight
